# Real world energy and carbon costs of net-zero energy buildings

**DOI:** 10.1038/s44172-022-00009-4

**Published:** 2022-05-26

**Authors:** Miranda L. Vinay

**Affiliations:** Communications Engineering, https://www.nature.com/commseng

## Abstract

Findings from a recent publication in *Energy and Buildings* show that “net-zero energy” renovations can lead to net-positive energy buildings. But the results also raise concerns for the energy grid and overheating in the summer. The analysis of energy consumption of residential buildings give insight into future performance of a Dutch neighborhood’s deep energy-saving refurbishments.

Humans spend energy in order to make their built environments comfortable, with buildings costing 40% of total energy demand in the EU and about 36% of EU carbon emissions^1^. Decarbonizing the Dutch building sector to hit climate targets requires over 200,000 buildings to be renovated annually with a mixture of energy-efficient and renewable energy generation solutions^2^. Before specific refurbishments can be recommended on a continental scale, the reductions in energy use and emission footprint must be adjusted to account for real systems – the people who live there and the weather they experience. In other words, do “net-zero” buildings by design really produce as much energy as they consume? And moreover, are these buildings comfortable to occupy?

These questions, as well as understanding the impact of renovated buildings on the energy grid, were undertaken by Kazmi and coworkers. They analyzed the post-refurbishment energy demand of 35 identical buildings with identical net-zero energy renovations in Utrecht, Netherlands over 2019 and 2020. These dwellings were refurbished with façade insulation, air source heat pumps, and rooftop solar PV panels. Then, each home was fitted with several sensors for monitoring electricity demand, solar electricity generation, room temperature, and the energy demands and temperatures of various household appliances. The acquired data was then combined with weather data from DarkSky and the data of the CO_2_ content of the electricity mix from ElectricityMap. Thermal comfort for residents was a focus of this work and a building exceeding an internal temperature of 28 °C was considered to be overheated. The result is a highly-detailed account of individual households and their cumulative consequences for resilience in the face of climate change and energy demand on the grid.

After two years, the researchers found the households to be emissions-positive for environmental impact with the neighborhood emitting around 150 kg of emissions per household per year and offsetting around 225 kg. Energy consumption, however, varied much more across individual households. “Most buildings turned out to be positive energy. This led us to the conclusion that designing net-zero neighborhoods should be preferable to designing net-zero buildings to account for the uncertainty caused by occupant behavior (and to alleviate grid load)” said Dr. Kazmi of the findings. The energy demand ranged between 2.5–7 megawatt hours per annum (MWh/a) and energy generation ranged between 5.5–8.5 MWh/a. However, despite the annual surplus generation, low solar irradiance during Dutch winters means that solar energy generation will not be enough to handle the weather-induced demand during cold months.

Further, the data revealed that the building renovations often bring about a higher thermal inertia, potentially causing discomfort or distress for residents depending on weather conditions. Dr. Kazmi remarked, “… the single most important determinant of overheating in these buildings was prolonged heat waves, rather than isolated temperature peaks. This follows naturally from the high thermal inertia of the buildings, but also indicates that this must be taken into consideration when planning buildings that will be around for decades to come.” With an expected increase in mean temperature of 0.5 to over 2 °C by 2050 in the Netherlands, it is likely that climate change will exacerbate these heat waves, making them an important consideration for renovations to come.

Future moderations of habitat will need to consider environmentally-friendly and comfortable solutions in order to combat such climate change effects. The results from this study show the real integration of high-efficiency buildings which can guide radical climate-friendly adaptations for the European building stock over the upcoming decades. They conclude that such adaptions should only be considered for neighborhoods with sufficient grid capacity to prevent under-voltage events in the cold months and that care should be taken to prevent overheating. This should be seen as a more holistic design perspective of the future building stock than the current emphasis on energy efficiency—often at the expense of flexibility and resilience to climate change events both for the building and the grid itself.

When asked about the next steps, “the first is to develop a better understanding of the energy flexibility inherent in the building stock, and how the energy transition can be seen as an opportunity rather than a challenge.” said Dr. Kazmi. A net-zero energy future depends on an understanding of the real consequences of energy-efficient integration methods.

The original article can be found here: Kazmi, H. et al. Energy balances, thermal performance, and heat stress: Disentangling occupant behaviour and weather influences in a Dutch net-zero energy neighborhood. *Energy and Buildings* **263**, 112020 (2022); 10.1016/j.enbuild.2022.112020.
